# Letters to the Editor Comment on The Radiological Physics Center's standard dataset for small field size output factors (J Appl Clin Med Phys. 2012;13(5):282‐89)

**DOI:** 10.1120/jacmp.v15i2.4784

**Published:** 2014-03-06

**Authors:** Jakob B. Thomsen, Anders R. Beierholm, Kristian Boye, Jesper Carl

**Affiliations:** ^1^ Department of Medical Physics Oncology Aalborg University Hospital Aalborg Denmark; ^2^ Center for Nuclear Technologies DTU Risoe Campus Technical University of Denmark Roskilde Denmark

Dear Editor,

A standard dataset for small field output factors was published by the Radiological Physics Center (RPC).^(1)^ The data are to be used as part of the quality control in other radiotherapy departments. As part of commissioning a new linear accelerator, we performed a comparison of our data against the original RPC dataset and found relative large deviations. We, therefore, conducted a comparison of the RPC dataset with measurements performed at four oncology departments in Denmark in order to validate the RPC dataset. The measurements were performed on Varian TrueBeam accelerators (Varian Medical Systems, Palo Alto, CA) using three different detectors all suited for small field dosimetry: a pinpoint chamber (PTW 31014; PTW, Freiburg, Germany), a prototype scintillator system developed at Technical University of Denmark,^(2)^ and a diamond detector (PTW 60003). In addition we included some data measured on a Varian Clinac 2100C/D accelerator. Furthermore, we included output factors calculated by the treatment planning systems of the participating centers.

Comparing our data to the original RPC dataset revealed a significant difference for all energies and a difference in output factors of about 2.5%. Recently an error in the original dataset was discovered, which lead to a major change in the output factors (see Followill, et al.: Erratum; Vol.15 #1).

In 1, 4, a plot of all measured and calculated values is seen for 6, 10, 15, and 18 MV beam qualities, respectively. All RPC data are taken from the corrected dataset and the error

**Figure 1:**
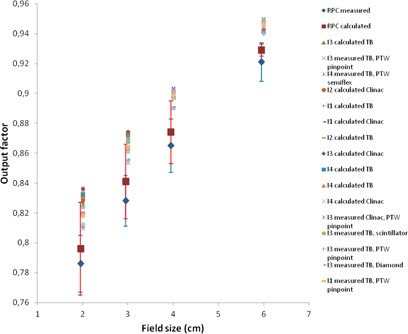
Plot of 6 MV output factors. For clarity, the RPC data are shown with a small offset in field size. Institutions are abbreviated I1‐I4; TB = TrueBeam.

bars represent one standard deviation. For 6 MV, we observe that the RPC measurements for all field sizes are lower than any of our measurements. To investigate further whether our data differ significantly from the RPC data, an analysis of variance (ANOVA) was performed using the statistical software STATISTICA (StatSoft Inc., Tulsa, OK). The measured RPC values were compared to the measured values at the participating centers with site, field size, detector type, and energy as variables. The ANOVA analysis determined a significant intercept of 0.0036 indicating an overall significant difference between the two datasets. Field size is not a significant variable, and we obtain F=0.34(p=0.80). Significant differences were obtained for site F=5.46(p=0.001), for detector F=2.92(p=0.037), and for energy $F=27.62(p<10^{-5})$. For 6 MV, the ANOVA estimated the output factors at the Danish centers to be larger than RPC data by a value of 0.0103 (about 1.2%), with a 95% confidence interval of [0.0084; 0.0123]. For 10 MV, the difference is 0.0041 (about 0.5%). For 15 and 18 MV, the output factors are not significantly different from the RPC dataset.

**Figure 2:**
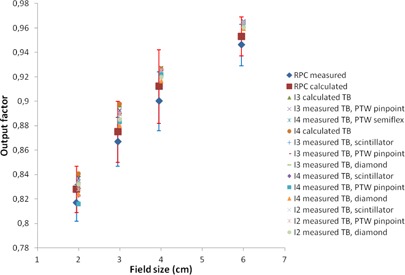
Plot of 10 MV output factors. For clarity, the RPC data are shown with a small offset in field size. Institutions are abbreviated I1‐I4; TB = TrueBeam.

**Figure 3:**
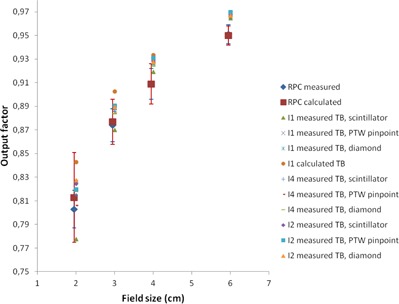
Plot of 15 MV output factors. For clarity, the RPC data are shown with a small offset in field size. Institutions are abbreviated I1‐I4; TB = TrueBeam.

**Figure 4:**
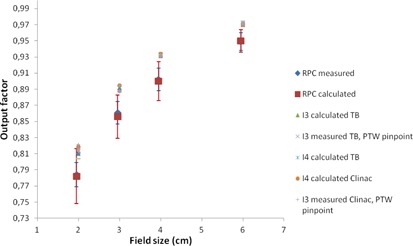
Plot of 18 MV output factors. For clarity, the RPC data are shown with a small offset in field size. Institutions are abbreviated I1‐I4; TB = TrueBeam.

We conclude that for 6 MV and 10 MV, there seem to be significant differences between our measurements and the corrected data published by the RPC. Although the corrections applied to the RPC dataset have improved the compliance with our data, great caution must be advised if using the RPC dataset as a reference during commissioning of Varian TrueBeam accelerators.
